# Minimal clinically important difference (MCID), substantial clinical benefit (SCB), and patient-acceptable symptom state (PASS) in patients who have undergone total knee arthroplasty: a systematic review

**DOI:** 10.1186/s43019-024-00210-z

**Published:** 2024-01-11

**Authors:** Filippo Migliorini, Nicola Maffulli, Luise Schäfer, Francesco Simeone, Andreas Bell, Ulf Krister Hofmann

**Affiliations:** 1 Department of Orthopaedic, Trauma, and Reconstructive Surgery, RWTH University Medical Centre, Pauwelsstraße 30, 52074 Aachen, Germany; 2 Department of Orthopedics and Trauma Surgery, Academic Hospital of Bolzano (SABES-ASDAA), Teaching Hospital of the Paracelsus Medical University, 39100 Bolzano, Italy; 3 Department of Trauma and Orthopaedic Surgery, Faculty of Medicine and Psychology, University la Sapienza, 00185 Rome, Italy; 4 School of Pharmacy and Bioengineering, Keele University Faculty of Medicine, Stoke On Trent, ST4 7QB UK; 5 Centre for Sports and Exercise Medicine, Barts and the London School of Medicine and Dentistry, Mile End Hospital, Queen Mary University of London, London, E1 4DG UK; 6 Department of Orthopaedic and Trauma Surgery, Eifelklinik St.Brigida, 52152 Simmerath, Germany

**Keywords:** Knee, Arthroplasty, Patient-reported outcome measures, PROMs, MCID, SCB, PASS

## Abstract

**Background:**

The present systematic review investigated the minimal clinically important difference (MCID), substantial clinical benefit (SCB), and patient-acceptable symptom state (PASS) of several frequent and established PROMs used to assess patients who have undergone TKA. This study was conducted according to the 2020 PRISMA statement.

**Methods:**

In September 2023, PubMed, Web of Science, and Embase were accessed with no time constraint All clinical studies investigating tools to assess the clinical relevance of PROMs used to evaluate patients having received TKA were accessed. Only studies which evaluated the MCID, PASS, or SCB were eligible. The PROMs of interest were the Forgotten Joint Score-12 (FJS-12), the Oxford Knee Score (OKS), the Knee Injury and Osteoarthritis Outcome Score (KOOS) and its related subscales activity of daily living (ADL), pain, quality of life (QoL), sports and recreational activities, and symptoms, the Western Ontario and McMaster Universities Osteoarthritis (WOMAC) score, the Knee Society Score (KSS) and related function score, and the Short Form-12 (SF-12) and Short Form-36 (SF-36).

**Results:**

Data from 29,737 patients were collected. The overall risk of bias was low to moderate. The great variability of thresholds for MCID, SCB and PASS between questionnaires but also between investigated aspects was noted, whereby MCIDs for the SF-36 appear lower than for knee-specific questionnaires.

**Conclusion:**

Despite its critical role from a patient’s perspective, the dimension of SCB is still neglected in the literature. Moreover, thresholds for the different concepts need to be condition-specific. We encourage authors to specifically report such data in future studies and to adhere to previously reported definitions to allow future comparison.

*Level of evidence* Level IV, systematic review and meta-analysis

## Introduction

Knee osteoarthritis (OA) affects large parts of the elderly population and is one of the leading causes of disability worldwide [1]. OA leads to reduced flexibility and mobility of the joint, and to load-dependent joint pain that can severely disable the patient, resulting in a high socio-economic burden [2]. If non-operative management of OA fails, partial or total knee arthroplasty (TKA) is performed. The affected joint surface is thereby replaced with prosthesis components to restore pain-free movement. While this highly standardised procedure achieves good to excellent results in most patients, 10–20% of them report persistent knee pain and functional limitations [3]. Great efforts are, therefore, still made to further improve outcomes after TKA.

Recent developments include the use of navigation and robotics [4], preoperative 3-dimensional planning [4, 5], the use of surface and insert geometries which allow more physiological joint kinematics [6–8] and the introduction of new alignment strategies that focus on the soft tissue needs than previous philosophies based solely on mechanical alignment [9].

The long-term effects of these new approaches may take up decades to become appreciable from registry data [4]. Patient satisfaction with the procedure, and the key dimension of success, is usually evaluated using patient-reported outcome measures (PROMs) [10]. Meaningful results can already be obtained during the first operative year, and a relatively steady state may be expected thereafter [11, 12]. PROMs are thus essential to evaluate the performance of TKAs and compare the performance of new techniques against established standards. When evaluating therapeutic success, a critical component of reporting medical data is the use of a p-value cut-off point of 0.05 [13]. In the scientific literature, results are routinely categorised as being either statistically significant or not significant [14]. Signalling the probability of error concerning a null hypothesis is a simple tool to characterise data and their potential relevance. This method, however, carries several pitfalls, which might be underrated in a clinical setting. First, clinically relevant differences might be missed because of false negative results (type II error) or falsely positively interpreted in case of a positive result (type I error), the likelihood of both being higher in underpowered studies [15, 16]. Second, even very small differences of no clinical relevance may reach a *p* < 0.05 when the sample size is simply large enough. Statistical significance, however, does not imply clinical relevance. Believing that statistically significant results always imply a clinically relevant finding can entail an erroneous application of study results [17].

To better interpret the clinical impact of study findings, new criteria have been proposed [18]. When using PROMS, minimum thresholds can be determined that still represent a clear or important benefit. Jaeschke et al. proposed the term “minimal clinically important difference (MCID)” (later also termed minimal important difference, MID) [19]. Acknowledging the relevance of such an approach, additional clinically oriented concepts have been introduced which can be used to better interpret PROM data. The MCID describes the smallest difference a patient can perceive in a specific questionnaire. The magnitude of a treatment-associated improvement that a patient recognises as a meaningful benefit is termed the substantial clinical benefit (SCB) [20]. The former two parameters are relative to the initial symptomatic state before treatment. A helpful concept to rate a cohort’s condition in absolute terms is the patient-acceptable symptom state (PASS), defined as the value on a PROM scale beyond which patients with a specific condition consider themselves well or in a satisfactory state [21]. Using all these parameters in the interpretation of study data, a better and patient-oriented description of obtained success rates in therapeutic approaches can be provided.

The results of surgical procedures depend on numerous factors. In the case of TKA, this is certainly the surgery itself, but also patient expectations before surgery, the degree of improvement in knee function and pain relief and potentially also socioeconomic domains [3]. Therefore, parameters such as MCID, PASS, or SCB may need to be defined as condition-specific and possibly also context-specific. To date, reference values for these thresholds are scarce and scattered in the literature, and at the same time highly necessary to better interpret the findings arising from clinical studies. The present systematic review investigated the MCID, PASS, and SCB of several frequent and established PROMs used to assess patients who have undergone TKA.

## Methods

### Eligibility criteria

All clinical studies investigating tools to assess the clinical relevance of PROMs used to assess patients having received TKA were accessed. Only studies which evaluated the MCID, PASS, or SCB were eligible. According to the authors’ language capabilities, articles in English, German, Italian, French, and Spanish were eligible. Only studies with levels I to IV of evidence, according to the Oxford Centre of Evidence-Based Medicine [22], were considered. Reviews, opinions, letters, and editorials were not considered. Missing quantitative data under the outcomes of interests warranted the exclusion of the study.

### Search strategy

This study was conducted according to the Preferred Reporting Items for Systematic Reviews and Meta-Analyses: the 2020 PRISMA statement [23]. The PICOD algorithm was preliminarily established:

- P (Problem): Endstage knee OA;
- I (Intervention): TKA;
- C (Comparison): Tool to assess the clinical efficacy of surgery;
- O (Outcomes): MCID, PASS, SCB;
- D (Design): Clinical study.

In September 2023, the following databases were accessed: PubMed, Web of Science, and Embase. No time constraint was set for the search. The Medical Subject Headings (MeSH) used for the database search are reported in the Appendix. No additional filters were used in the database search.

### Selection and data collection

Two authors (****) independently performed the database search. All the resulting titles were screened by hand and, if suitable, the abstract was accessed. The full text of the abstracts which matched the topic of interest was accessed. If the full text was not accessible or available, the article was not considered for inclusion. A cross reference of the bibliography of the full-text articles was also performed for inclusion. Disagreements were debated and mutually solved by the authors. In case of further disagreements, a third senior author (**) made the final decision.

### Data items

Two authors (****) independently performed data extraction. The following data at baseline were extracted: author, year of publication, country, study design, journal, type of analysis performed, type of PROMs investigated, and number of patients included. Data on the MCID, PASS, and SCB were collected. The PROMs of interest were the Forgotten Joint Score-12 (FJS-12) [24], the Oxford Knee Score (OKS) [25], the Knee Injury and Osteoarthritis Outcome Score (KOOS) and its related subscales activities of daily living (ADL), pain, quality of life (QoL), sports and recreational activities, and symptoms [26], the Western Ontario and McMaster Universities Osteoarthritis (WOMAC) score [27, 28], the Knee Society Score (KSS) and related function score [29], and the Short Form-12 (SF-12) [30] and Short Form-36 (SF-36) [30–33]. Data were extracted in Microsoft Office Excel version 16.72 (Microsoft Corporation, Redmond, USA).

### Methodological quality assessment

The risk of bias was evaluated following the guidelines in the Cochrane Handbook for Systematic Reviews of Interventions [34]. Two reviewers (****) evaluated the risk of bias in the extracted studies. The Risk of Bias in Nonrandomised Studies of Interventions (ROBINS-I) tool was used [35]. The tool is completed in three phases. During phase one, the relevance of the research question is evaluated (optional). Phase two considers four domains through which risks of bias can be introduced into systematic reviews: study eligibility criteria, identification and selection of studies, data collection, summary, and results. Phase three assesses the overall risk of bias in the interpretation of the review findings, and whether the interpretation has taken into account any limitations identified in any of the domains of phase two. Seven domains of potential bias in non-RCTs were assessed. Possible confounding and the nature of patient selection before the start of the comparative intervention are assessed by two domains. A further domain is used to assess bias in the classification during the intervention. The final four domains assess the methodological quality after the start of the intervention: biases from deviations from originally intended interventions, missing data, erroneous measurement of outcomes, or bias in the selection of the reported outcomes are evaluated. The figure of the ROBINS-I was elaborated using the Robvis Software (Risk-of-bias VISualization, Riskofbias.info, Bristol, UK) [36].

### Synthesis methods

The statistical analyses were performed by the main author (**) following the recommendations of the Cochrane Handbook for Systematic Reviews of Interventions [37]. For descriptive statistics, the arithmetic mean was used using the IBM SPSS software version 25 (International Business Machines Corporation, Armonk, USA).

## Results

### Study selection

**Fig. 1:**
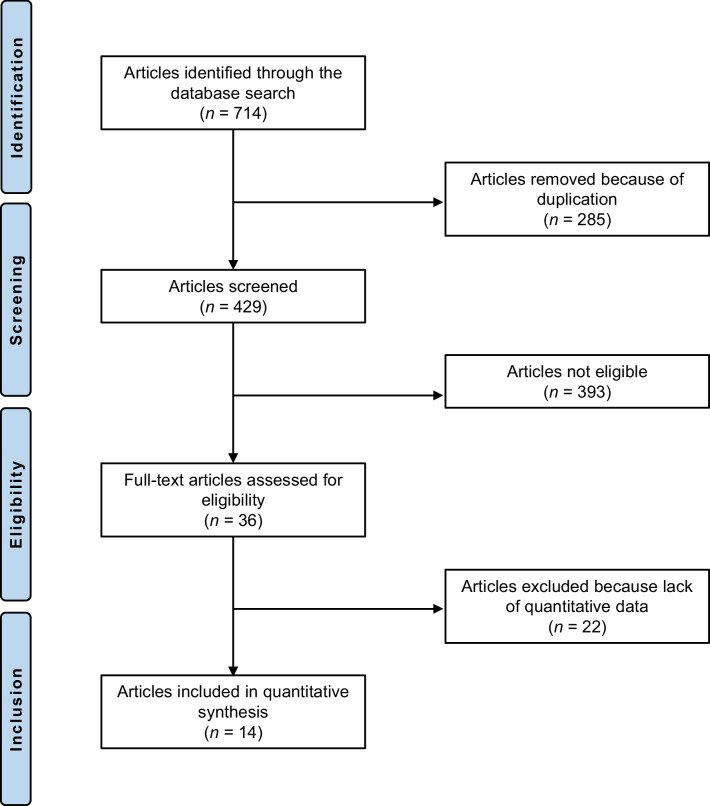
Flow chart of the literature search

The systematic literature search resulted in 714 articles. A total of 285 were identified as duplicates and, therefore, excluded. A further 393 investigations were discarded as they did not meet the predefined inclusion criteria. The reasons for exclusion were inadequate study design (*N* = 291), low level of evidence (*N* = 17), missing implementation of at least one tool to determine the clinical relevance of PROMs (MCID, PASS, or SCB) (*N* = 31), not reporting data from at least one PROM of interest (FJS-12, OKS, KOOS, WOMAC, KSS, SF-12 or SF-36) (*N* = 35), and language limitations (*N* = 9). After full-text evaluation, an additional 22 investigations were excluded because they did not offer any quantitative data on the outcomes of interest. In conclusion, 14 studies were available for inclusion. All of them were non-RCTs. The results of the literature search are shown in Fig. 1.

### Methodological quality assessment

**Fig. 2:**
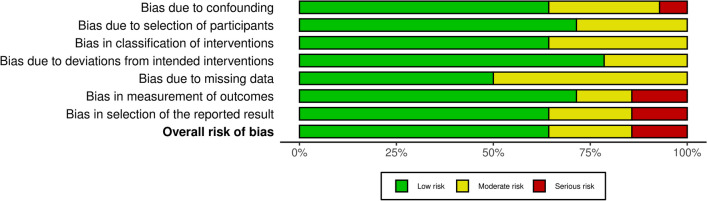
Risk of Bias in Nonrandomised Studies of Interventions (ROBINS-I) tool of the non-randomised clinical trials

The ROBINS-I was applied to investigate the risk of bias in all studies included in the present systematic review. Confounding could be ruled out in most articles. One study was rated with a serious risk of confounding, as there were differences in the intervention groups at baseline. Patient selection was described in detail in all studies. Exclusion of patients or differences in follow-up time of individual patients were predominantly not detected, which indicates an overall low to moderate risk of bias based on participant selection. The risk of bias in the classification of interventions resulted mainly low, as there were neither nondifferential misclassification nor differential misclassification identified. Furthermore, systematic differences between the experimental and comparison groups were not found, leading to a low to moderate risk of deviations from the intended interventions. The lack of assessor blinding in all studies reviewed indicated a predominantly moderate risk of bias in the measurement of outcomes. Given the mainly good quality of the included studies, the overall risk of bias was low to moderate. The ROBINS-I is reported in Fig. 2.

### Study characteristics and results of individual studies

Data from 29,737 patients were collected. The generalities and demographics of the included studies are shown in Table 1.

**Table 1:** Generalities and patient baseline of the included studies

Author	Year	Country	Design	Journal	Analysis	PROMs	Patients (n)
Ayers et al. [38]	2023	USA	Retrospective	J Arthroplasty	MCID	KOOS	11,602
Carender et al. [39]	2022	USA	Retrospective	J Arthroplasty	MCID/PASS/SCB	WOMAC/ KOOS	8,600
Clement et al. [40]	2014	Scotland	Prospective	Knee Surg Sports Traumatol Arthrosc	MCID	OKS/ SF-12	505
Clement et al. [41]	2018	UK	Retrospective	Clin Orthop Relat Res	MCID	WOMAC	2,589
Clement et al. [42]	2021	UK	Prospective	Bone Joint J	MCID	FJS-12	484
Clement et al. [43]	2022	UK	Retrospective	Bone Joint Res	MCID/PASS	SF-36 PF	3,791
Escobar et al. [44]	2007	Spain	Prospective	Osteoarthritis Cartilage	MCID	WOMAC/ SF-36	65
Escobar et al. [45]	2013	Spain	Prospective	Osteoarthritis Cartilage	MCID	WOMAC	415
Gousopoulos et al. [46]	2023	France	Retrospective	Knee Surg Sports Traumatol Arthrosc	PASS	OKS/ FJS-12	135
Heijbel et al. [47]	2022	Sweden	Retrospective	Acta Orthop	SCB	FJS-12	183
Ingelsrud et al. [48]	2021	Denmark	Retrospective	Acta Orthop	PASS	OKS	571
Lizaur-Utrilla et al. [49]	2020	Spain	Prospective	Knee Surg Sports Traumatol Arthrosc	MCID/SCB	KSS	507
Maxwell et al. [50]	2014	USA	Prospective	J Rheumatol	MCID/PASS	WOMAC	228
Nishimoto et al. [51]	2023	Japan	Prospective	J Orthop Trauma Rehab	MCID	KOOS	62

FJS-12: Forgotten Joint Score-12; KOOS: Knee injury and Osteoarthritis Outcome Score; KSS: Knee Society Score; MCID: minimal clinically important difference; OKS: Oxford Knee Score; PASS: patient-acceptable symptom state; PROMs: patient-reported outcome measures; SCB: substantial clinical benefit; SF: Short Form; WOMAC: Western Ontario and McMaster Universities Osteoarthritis

### Results syntheses

An overview of the results of MCID, PASS, and SCB about the FJS, OKS, KOOS, WOMAC, KSS, SF-12 and SF-36 is reported in Table 2.

**Table 2:** Main results

PROMs	Patients (*n*)	MCID	SCB	PASS
FJS-12 (0–100)	806	14	28	30
OKS (12–60)	1211	5		30
KOOS (0–100)	11,798	12		
KOOS ADL (0–100)	12,483	10	23	83
KOOS Pain (0–100)	12,314	12	22	85
KOOS QOL (0–100)	12,314	14	15	66
KOOS Sports/Recreation (0–100)	231	9		
KOOS Symptoms (0–100)	712	9	21	81
WOMAC function (0–20)	7524	24		67
WOMAC pain (0–68)	7558	24		75
WOMAC stiffness (0–8)	2654	15		
WOMAC total (0–96)	2589	10		
KSS (0–100)	701	5	40	
KSS function (0–100)	507	10	39	
SF-12 (0–100)	701	6		
SF-36 bodily pain (0–100)	3856	7		54
SF-36 mental health (0–100)	3856	4		69
SF-36 physical functioning (0–100)	3856	7		34
SF-36 role-emotional (0–100)	3856	2		65
SF-36 social functioning (0–100)	3856	7		56
SF-36 vitality (0–100)	3856	3		47
SF-36 role physical (0–100)	3856	9		43
SF-36 total (0–100)	3856	5		51

ALD: activity of daily living; FJS-12: Forgotten Joint Score-12; KOOS: Knee injury and Osteoarthritis Outcome Score; KSS: Knee Society Score; MCID: minimal clinically important difference; OKS: Oxford Knee Score; PASS: patient-acceptable symptom state; PROMs: patient-reported outcome measures; QoL: quality of life; SCB: substantial clinical benefit; SF: Short Form; WOMAC: Western Ontario and McMaster Universities Osteoarthritis). In brackets the range of points each scale offers

## Discussion

This study systematically investigated the MCID, PASS, and SCB of several frequent and established PROMs used to assess patients who have undergone TKA. Fourteen studies were eventually analysed including 29,737 patients.

When examining the different magnitudes of MCIDs in the various questionnaires, a relatively large variability between questionnaires can be observed. Very low MCIDs were reported for the different dimensions of the SF-36 (most values clearly below 10). In contrast, in the knee-specific questionnaires, the MCID was much greater, with mostly values clearly above 10, and in the case of the WOMAC, even above 20 in some instances. For KOOS-ADL, -Pain, and -QoL values were almost identical for TKA, for example, compared to chondral procedures of the knee. In contrast, values for the dimensions of sports/recreational activities and symptoms were much lower for TKA than for cartilage repair procedures [52–54]. The reason for this phenomenon can only be speculated upon. One possible explanation might be that values for sports/recreational activities might be already so low in patients scheduled for TKA that even minor improvements exert a strong impact on the patient’s perception of the condition. This is in contrast to cartilage repair procedures, where a relatively high activity level is present preoperatively. Of note, the MCIDs for the KOOS (total score) described in the present review are in agreement with those reported in a systematic review displaying both distribution and anchor-based derivation of MCIDs [55]. Their study included 18 studies—two of which were included in the present investigation.

Although probably the most important parameter from a patient’s perspective, the SCB was rarely evaluated in the included studies. In the FJS-12 and the KOOS, values were, however, twice as high for the SCB than for the MCID except QoL (where MCID and SCB were almost identical). In the KSS, SCB values were even 4 times higher for the MCID. This illustrates the fact that both parameters—MCID and SCB—have their justification and that a single presentation of an improvement at the size of the MCID does not necessarily imply a sufficient improvement for the patient. SCB levels after TKA were also calculated by Lyman et al. [56], who reported a range from 15 to 36 for the different KOOS dimensions. Interstingly, Haydel et al. observed in a cohort of TKA that better preoperative KOOS-Symptoms, -QoL, and -ADL living subscale scores were statistically significantly associated with failing to meet the MCID and SCB on each respective subscale [57].

For the PASS, a great variability between questionnaires but also between investigated aspects was noted. In the SF-36, for example, the PASS scoring for mental health was over twice as high (69) than for physical functioning (34). Values for a PASS were highest in the KOOS with QoL (83) and Pain (85), whereas they were low in the FSJ-12 with only 30 of 100 points. This low value for the FSJ-12 (33.3) was, however, also reported by Singh et al. 2022 [58], using a receiver operating characteristic curve point to calculate the value. In another study, the PASS in the KOOS ranged from 80 to 88 except QoL with 66 points [12]. Similar to the MCID, PASS values seem to be strongly dependent on the condition they are applied to. In a recent systematic review, PASS thresholds in KOOS-ADL for ACL tears were 92 to 100, and KOOS-Symptoms (73–78) and KOOS-QoL (53–57) in meniscus injuries [59].

When interpreting PASS values, it needs to be considered from which baseline values in questionnaires patients started. A retrospective registry study observed that patients suffering from OA and treated with conservative means defined lower PASS values post-intervention when they also had lower baseline values [60]. Caution also needs to be exercised when relating to PASS values reported in the literature. While for MCID and SCB it is somehow clear that threshold values are discussed, for the PASS it is often the rate of patients having achieved such a symptom state that is related to (e.g. [61]). Such a rate can be calculated as a ratio of patients having met a certain threshold [61], the universal definition of which is still a matter of debate as can be seen by the data presented in this systematic review. Alternatively, a PASS is directly evaluated simply by anchor questions. Depending on the wording, chosen increments and context of these anchor questions, completely different results might be obtained.

The limitations of the present study are mostly related to the low number of available studies reporting quantitative data on MCID, SCB, and PASS in the context of TKA (*n* = 14). While at least the number of patients included is quite substantial in some of these studies, not all values were reported for all three values in all questionnaires. The lack of a mix of data from several studies per item makes the presented available data prone to selection, recruitment or reporting bias. Moreover, it was not specifically reported whether the data presented were derived by distribution or anchor-based methods. The mode of data derivation influences the thresholds calculated, while although an anchor-based calculation may seemingly be more intuitive, it is of difficult application, especially in retrospective studies when the necessary anchor questions are missing [39, 55]. Distribution methods, on the other hand, result in values that describe statistical significance and do not capture clinical changes as directly perceived by the patient [55]. For this reason, probably both derivation methods have their justification. Anchor-based techniques will, however, have to be standardised, and for both techniques, their independent threshold values will need to be established.

Despite these imprecisions, we are convinced of the concept of judging PROM data on their clinical relevance by applying MCID, SCB, or PASS. We encourage authors to specifically report such data in future studies and to adhere to previously reported definitions to allow future comparison.

## Conclusion

We found substantial variability of thresholds for MCID, SCB and PASS between questionnaires but also between investigated aspects. Thresholds thus need to be condition-specific in patients undergoing TKA. Although clinically important, SCB is still neglected in the literature. We encourage authors to report such data in future studies and to adhere to previously reported definitions to allow future comparison.

## Data Availability

The datasets generated during and/or analysed during the current study are available throughout the manuscript.

## Abbreviations

ADL: Activities of daily living
FJS-12: Forgotten Joint Score-12
KOOS: Knee Injury and Osteoarthritis Outcome Score
KSS: Knee society score
MCID: Minimal clinically important difference
MID: Minimal important difference
OA: Osteoarthritis
OKS: Oxford knee score
PASS: Patient-acceptable symptom state
PRISMA: Preferred Reporting Items for Systematic Reviews and Meta-Analyses
PROM: Patient-reported outcome measure
QoL: The quality of life
RCT: Randomised controlled trial
SCB: Substantial clinical benefit
SF: Short form
TKA: Total knee arthroplasty
WOMAC score: Western Ontario and McMaster Universities Osteoarthritis score
